# Management and clinical analysis of complications following all-on-4 implant-supported rehabilitation in a patient with severe maxillary bone deficiency and periodontitis: a case report

**DOI:** 10.1186/s12903-026-08944-w

**Published:** 2026-06-16

**Authors:** Xun Xia, Chang-Qi Hu, Jiang-Qin Huang, Hong-Wu Wei

**Affiliations:** 1 People’s Hospital of Pingshan Shenzhen, Pingshan Hospital of Southern Medical University, Shenzhen, Guangdong Province 518000 China; 2 Fourth Affiliated Hospital of Nanchang University, Nanchang, Jiangxi Province 330000 China

**Keywords:** All-on-4 implant technique, Parkinson's disease, Chronic periodontitis, Dentition defect, Maxillary sinus floor elevation, Implant complications

## Abstract

**Background:**

The All-on-4 implant technique offers an effective rehabilitation solution for maxillary bone - deficient patients, yet preventing and controlling complications in those with severe periodontitis and Parkinson’s disease (PD) poses a clinical challenge.

**Case presentation:**

A 63-year-old PD patient had multiple teeth extracted due to mobility years ago and a history of failed implant rehabilitation in the posterior maxilla at another hospital. He was diagnosed with dentition defect and chronic periodontitis upon admission. Immediate All-on-4 implant rehabilitation was performed after extraction of the remaining maxillary teeth. Three months postoperatively, implant at site 15 became loose and was removed; three months later, tilted implantation was performed at site 15, and simultaneous maxillary sinus floor elevation with implant placement was conducted at sites 16 and 17. Six months after the second operation, implant 25 failed and was extracted; two months later, tilted implantation was re-performed at site 25, and simultaneous maxillary sinus floor elevation with implant placement was carried out at sites 26 and 27. Final fixed prosthodontics was completed, and during the 55-month follow-up, the prosthesis has remained stable, and masticatory function has been significantly improved compared with the pre-treatment status.

**Conclusion:**

This case report firstly presents a complication management protocol following All-on-4 rehabilitation in patients with PD. For PD patients complicated with severe maxillary bone deficiency and concurrent periodontitis, All-on-4 rehabilitation mandates rigorous preoperative periodontal treatment, individualized implant site design, reliable patient compliance, and standardized periodic postoperative maintenance. Dentists should stay alert to potential obstacles to oral self-care and xerostomia in PD individuals.

## Introduction

The All-on-4 implant technique involves placing four implants with a tilted and axial design. This approach avoids complex bone augmentation procedures, facilitates primary stability, enables immediate rehabilitation, and significantly shortens the treatment cycle [1]. It has thus become one of the preferred rehabilitation options for both dentists and patients. However, the incidence of peri-implantitis is significantly higher in patients with chronic periodontitis, directly affecting the long-term survival rate of implants [2]. On the other hand, the All-on-4 technique imposes high requirements on both dentists and patients; failure of more than one implant may preclude the completion of final prosthodontics.

Literature reports indicate that the incidence of Parkinson’s disease (PD) is increasing year by year, with a global pooled prevalence of 1.51 cases per 1,000 (95% CI 1.19–1.88) [3], which implies that the current global number of affected individuals exceeds 10 million. Patients with PD often suffer from xerostomia, with a survey revealing that among 111 PD patients, the incidence of xerostomia was 48.6%, compared to 22.1% in the control group [4]. In addition to salivary flow dysregulation caused by autonomic dysfunction in PD patients, commonly used therapeutic agents such as anticholinergics and dopaminergics can also induce or exacerbate xerostomia as an adverse effect [5]. Saliva plays a crucial role in maintaining oral health, including lubricating oral mucosa, clearing food debris, neutralizing acidic substances, exhibiting antibacterial properties, and promoting tooth remineralization. When salivary secretion decreases, the self-cleaning and protective mechanisms of the oral cavity are compromised, thus increasing the risk of various oral diseases and even threatening the health of dental implants [6, 7]. Studies have shown that patients with PD commonly exhibit issues such as impaired ability to perform toothbrushing, poor oral hygiene, abnormal salivary secretion, and impaired oral motor function, all of which directly affect the success rate and long-term stability of dental implants [5].

Although case reports have documented favorable outcomes of All-on-4 rehabilitation in PD patients, detailed reports on complications associated with immediate All-on-4 rehabilitation in this patient cohort are currently scarce. This case report describes a patient with PD complicated by severe maxillary bone defect and chronic periodontitis, who achieved long-term treatment success after multiple implant failures through optimized therapeutic strategies. This case provides clinical insights for complication management in similar complex cases. By integrating relevant literature, this case report discusses the diagnostic and therapeutic challenges and optimized strategies for such complex patients, providing clinical reference.

## Case presentation

### Basic information

This study was conducted in accordance with the ethical guidelines of the World Medical Association as outlined in the 2013 Declaration of Helsinki. The patient’s information has been anonymized.

A 63-year-old retiree presented to the Department of Stomatology, the Fourth Affiliated Hospital of Nanchang University, in April 2019, complaining of “missing posterior maxillary teeth bilaterally for many years and poor performance of removable dentures, requesting fixed rehabilitation.” The patient was alert and able to communicate well. The patient has a 5-year history of PD and was evaluated by a specialist as stage 1.5 on the Hoehn-Yahr scale [8], with left-sided hemibody involvement accompanied by mild truncal symptoms such as truncal muscle pain and stiffness. Despite a significant decline in muscle strength, the patient retained the ability of daily living, brushing their teeth twice daily (morning and evening) for approximately 30 s each time. The patient’s PD was managed with oral benserazide + levodopa (Madopar, each tablet 0.25 mg, half a tablet twice daily) and pramipexole dihydrochloride (Sifro, each tablet 0.25 mg, half a tablet twice daily). The patient frequently complained of xerostomia and viscous saliva. Daily care measures for the patient included creating a safe home environment, wearing loose-fitting clothing, using a toothbrush with a thick handle for oral hygiene, engaging in 30 min of gentle exercise daily, and participating in relaxing daily activities such as drinking tea and playing chess with friends. No abnormalities in oral motor function were observed, but occasional hand tremors were noted. He denied a history of hypertension, heart disease, diabetes mellitus, or drug allergies. Several years prior, he had undergone extraction of posterior maxillary teeth due to severe periodontitis. He thus sought immediate fixed rehabilitation at our hospital.

### Physical examination

#### Extraoral examination

Facial symmetry was maintained, with a normal lower third facial height. No tenderness or crepitus was noted in the temporomandibular joint area, and masticatory muscle function was normal. The mouth opening degree was approximately 3 fingers (37 mm) with a vertical opening pattern. The patient had a median smile line, and no obvious abnormalities were observed in facial appearance.

#### Intraoral examination

**Fig. 1:**
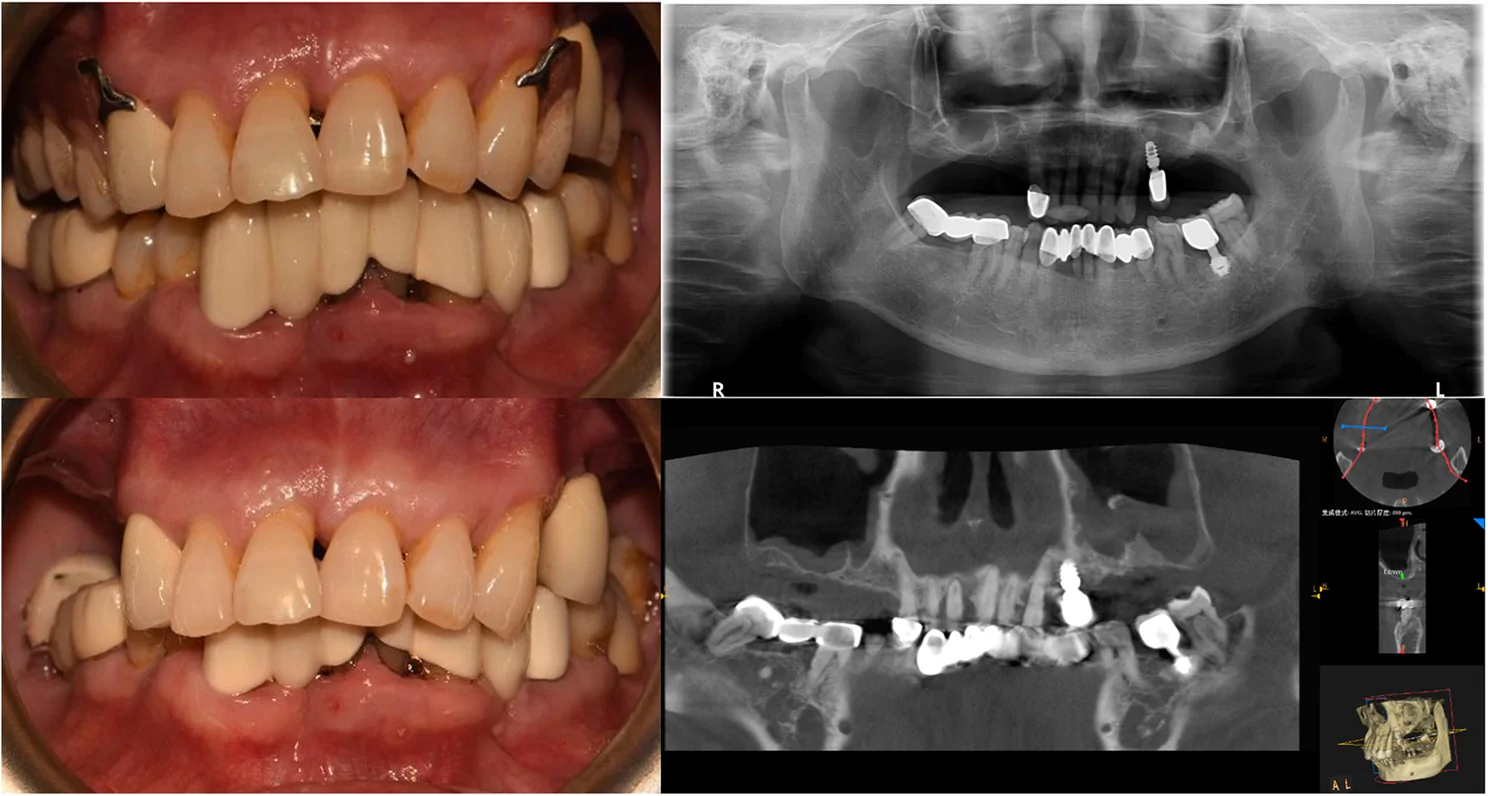
Intraoral examination and imaging examination

Oral hygiene was fair, with a full-mouth plaque index of 1, grade I dental calculus, significant gingival recession, and positive bleeding on probing (BOP). Posterior mandibular teeth were extruded, resulting in a deep overbite relationship between the maxilla and mandible, with a significantly accentuated Curve of Spee. Maxillary dentition examination revealed missing teeth at sites 17, 16, 15, 14, 24, 26, and 27. The alveolar ridge width in the edentulous areas was acceptable (5.2–9.3 mm), but significant loss of attached gingiva was noted. Teeth 11 and 21 were restored with porcelain-fused-to-metal crowns, showing extrusion and mobility of grade I–II, negative percussion, and positive BOP. No obvious mobility or percussion pain was observed in the remaining teeth (Fig. 1).

### Auxiliary examination

#### Laboratory examination

Preoperative routine blood tests, four coagulation function tests, liver function tests, and four pre-transfusion tests were all within normal ranges, excluding surgical contraindications.

#### Imaging examination

All images were taken at the intercuspal position and used Carestream CS 9300 Select (Carestream Health, Inc, France). Cone-beam computed tomography (CBCT) revealed poor overall periodontal tissue condition, with varying degrees of horizontal and vertical alveolar bone resorption. One width and height were measured by two independent dentists in a blinded manner, and the average value was taken. Adequate bone volume was observed in the anterior maxilla (sites 11, 21, 22, 23): site 11 had a bone width (W) of 7.0 mm and bone height (H) of 15.2 mm; site 21 had W = 9.3 mm and H = 14.0 mm; site 22 had W = 5.3 mm and H = 14.7 mm; site 23 had W = 8.4 mm and H = 12.4 mm. Severe bone deficiency was noted in the posterior maxilla: site 16 had H = 1.4 mm; site 17 had H = 1.2 mm; site 26 had H = 3.4 mm; site 27 had H = 1.5 mm; implant site 15 had H = 17.0 mm and W = 6.5 mm; implant site 25 had H = 13.7 mm and W = 8.0 mm (Fig. 1).

### Treatment

#### Preoperative periodontal treatment

Systematic oral hygiene education was provided to the patient, including guidance on the modified Bass brushing technique and floss use. Full-mouth supragingival scaling, subgingival scaling, and root planing were performed to control periodontal inflammation. A follow-up examination 1 week postoperatively showed significant improvement in gingival bleeding and a reduction in BOP-positive sites.

#### First-stage operation (April 21, 2019)

The treatment plan was designed as an immediate All-on-4 rehabilitation for the maxillary arch, with implants positioned at sites 15, 11, 21, and 25 based on the patient’s preference and oral conditions. The surgery was performed by Surgeon A with 10 years of experience in All-on-4 implant surgery, and all implants were placed using a freehand technique without a surgical guide. Thirty minutes before surgery, the patient received 0.3 g (1 capsule) of ibuprofen sustained-release capsule and 3 mg (4 tablets) of dexamethasone acetate orally. Under local anesthesia with articaine hydrochloride and epinephrine tartrate injection (4% articaine hydrochloride with 1:100,000 epinephrine; ‌Primacaine, Pierrel, Milan, Italy), the remaining maxillary natural teeth were extracted, and the implant 25 was removed by counterclockwise rotation. A full-thickness mucoperiosteal flap was elevated to expose the alveolar ridge crest. Granulation tissue was thoroughly debrided with a curette (Hu-Friedy, Chicago, USA), followed by copious irrigation with sterile saline. Bone rongeurs were used for alveolar ridge reduction, and the harvested autogenous bone was immersed in sterile saline for subsequent grafting. The osteotomy ensured a continuous alveolar ridge contour and a favorable prosthetic space of 10–15 mm. Site preparation was performed at 800 rpm with continuous cooling using 4 °C sterile saline. The initial drilling depth was 15–16 mm, and the final drill (diameter 4.3 mm) was set to approximately 8 mm. Four Nobel Active implants (Nobel Biocare, Sweden; diameter 4.3 mm, length 13 mm) were inserted with a motorized driver at an insertion torque of 35 Ncm, followed by final tightening with a manual torque wrench to reach an insertion torque of 40–45 Ncm. Based on CBCT measurements, the implant emergence profiles were located at sites 15, 11, 21, and 25. After implant placement, a shoulder osteotomy drill was used to contour the peri-implant bone. Multi-unit abutments were placed and tightened to 30 Ncm, followed by seating of multi-unit abutment protection caps. Autogenous bone was grafted into bone defects and jump gaps. Excess soft tissue was trimmed, and the wound was closed primarily. ‌A resin splint rigidly splinting four transfer copes was used for impression making, followed by the fabrication of an acrylic resin provisional fixed dental prosthesis. The gingival surface of the provisional prosthesis was highly polished and contoured to protrude toward the alveolar ridge. During provisional try-in, alternate digital pressure and the Sheffield test (University of Sheffield, School of Clinical Dentistry) were applied, accompanied by intraoral radiography to verify passive seating of the provisional multi-unit abutments. The provisional prosthesis was tightened to a torque of 15 Ncm.

**Fig. 2:**
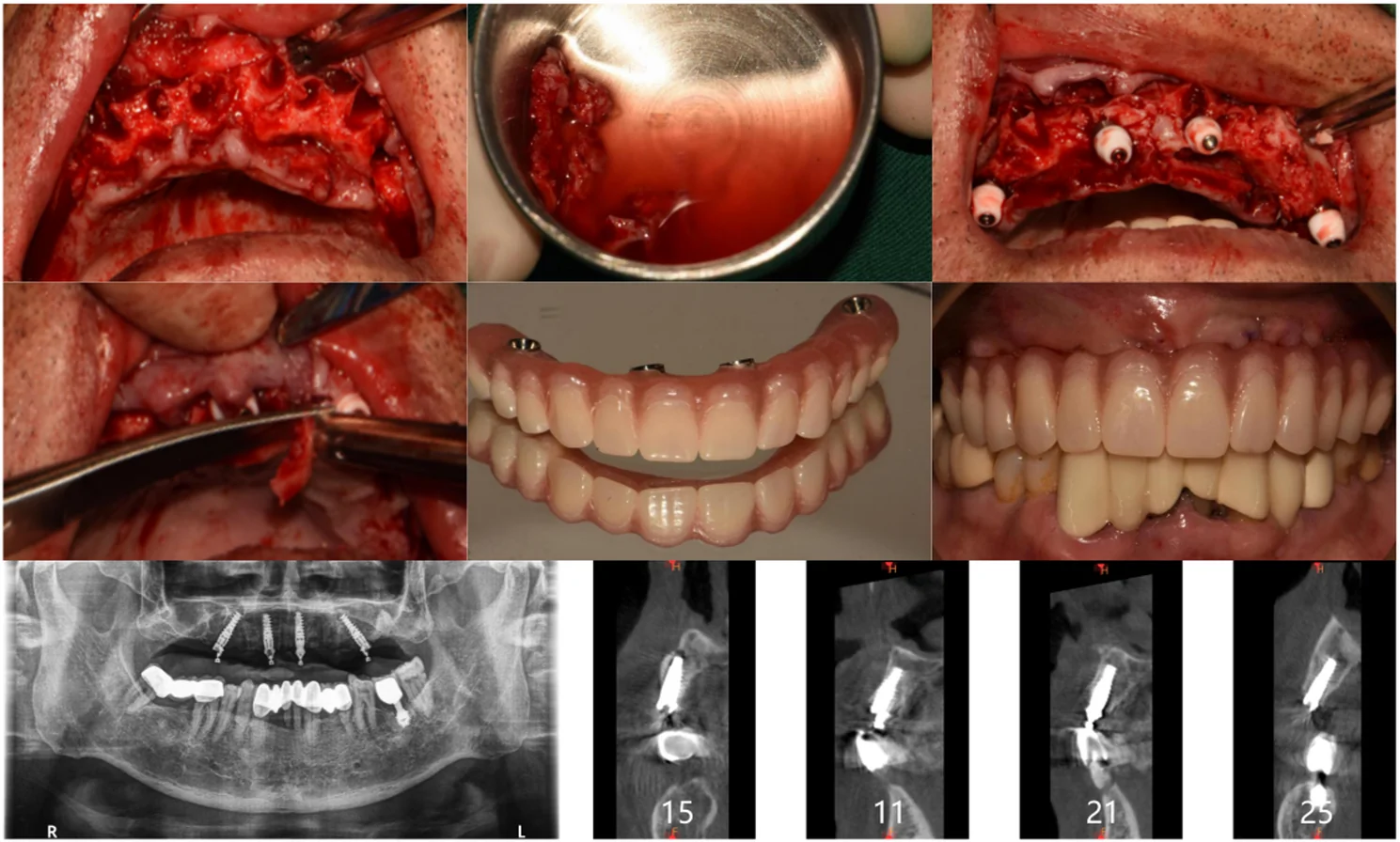
First-stage operation

Occlusal adjustment was performed to achieve broad bilateral contacts in centric occlusion, with shallow overbite and reduced overjet in the anterior region, and group function occlusion during lateral mandibular movements. CBCT evaluation confirmed an anterior-posterior (AP) distance of approximately 10 mm, and a 10-mm cantilever extension was designed at 16. The occlusal separation of the cantilever was adjusted to > 100 μm. Immediate temporary fixed prosthodontics was performed to restore basic masticatory function (Fig. 2). Postoperatively, antibiotics (amoxicillin combined with metronidazole) were prescribed for 3 days for infection prophylaxis, dexamethasone acetate was administered for 3 days to control postoperative edema, and compound chlorhexidine gluconate mouth rinse was recommended for 7 days. The patient was instructed to avoid hard foods and maintain a mainly soft and liquid diet. In addition, the patient was advised to pay close attention to oral hygiene, clean all tooth surfaces thoroughly, brush teeth for approximately 3 min each time, and use an oral irrigator twice daily after meals.

#### Management of first implant failure (July 20, 2019)

**Fig. 3:**
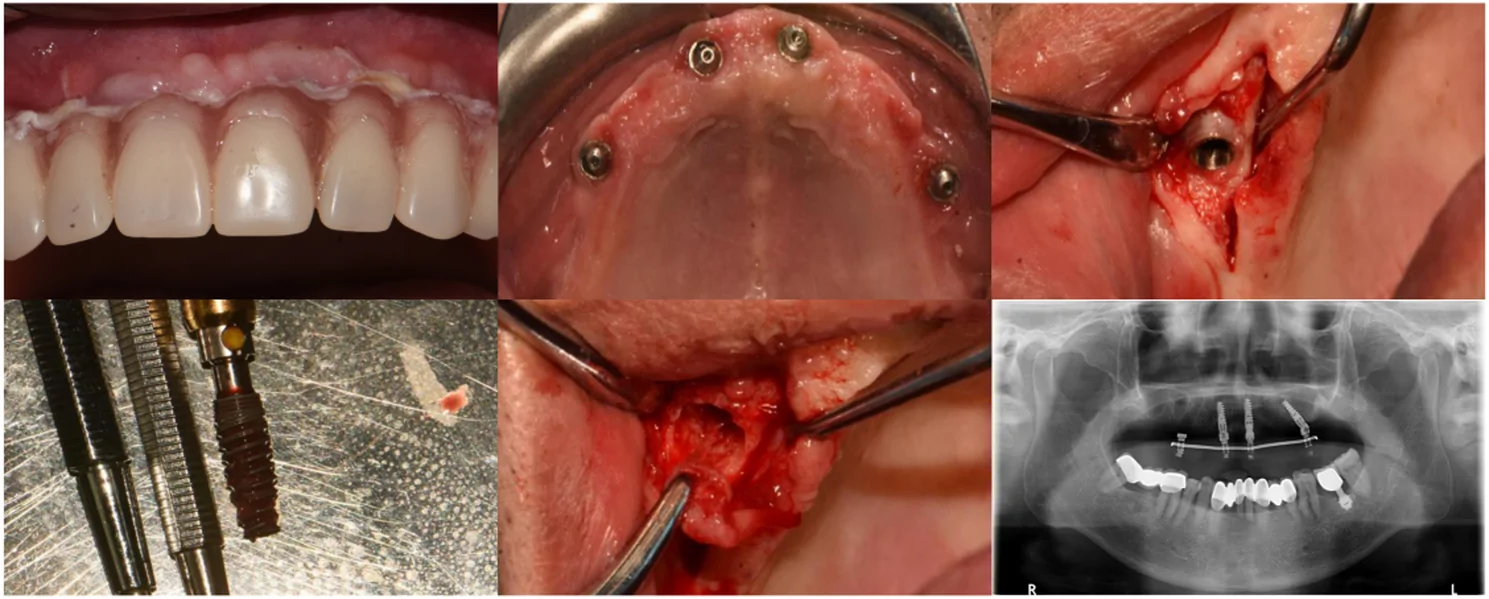
Management of first implant failure

The patient exhibited poor oral hygiene maintenance. A substantial amount of plaque and debris was detected between the provisional prosthesis and the gingiva, and redness and swelling were observed in some regions of the gingiva. A follow-up clinical examination conducted 3 months postoperatively demonstrated significant loosening of implant at site 15 (mobility > 2 mm), leading to the diagnosis of implant failure. Under local anesthesia, flap elevation surgery was performed to remove the failed implant; subsequently, the implant socket was thoroughly debrided with a curette, and the wound was sutured following copious irrigation with sterile saline. Given the patient’s esthetic requirements, the cantilever extending from sites 11–16 was retained, with a total length of approximately 25 mm (Fig. 3). The cantilever segment was adjusted and ground to achieve an occlusal separation greater than 100 μm. The patient was instructed to restrict mastication to the left side and adhere to a soft or liquid diet. Special emphasis was placed on the importance of enhancing oral hygiene maintenance to prevent complications. The postoperative medication regimen was consistent with that administered during the first-stage operation.

#### Second-stage operation (November 22, 2019)

**Fig. 4:**
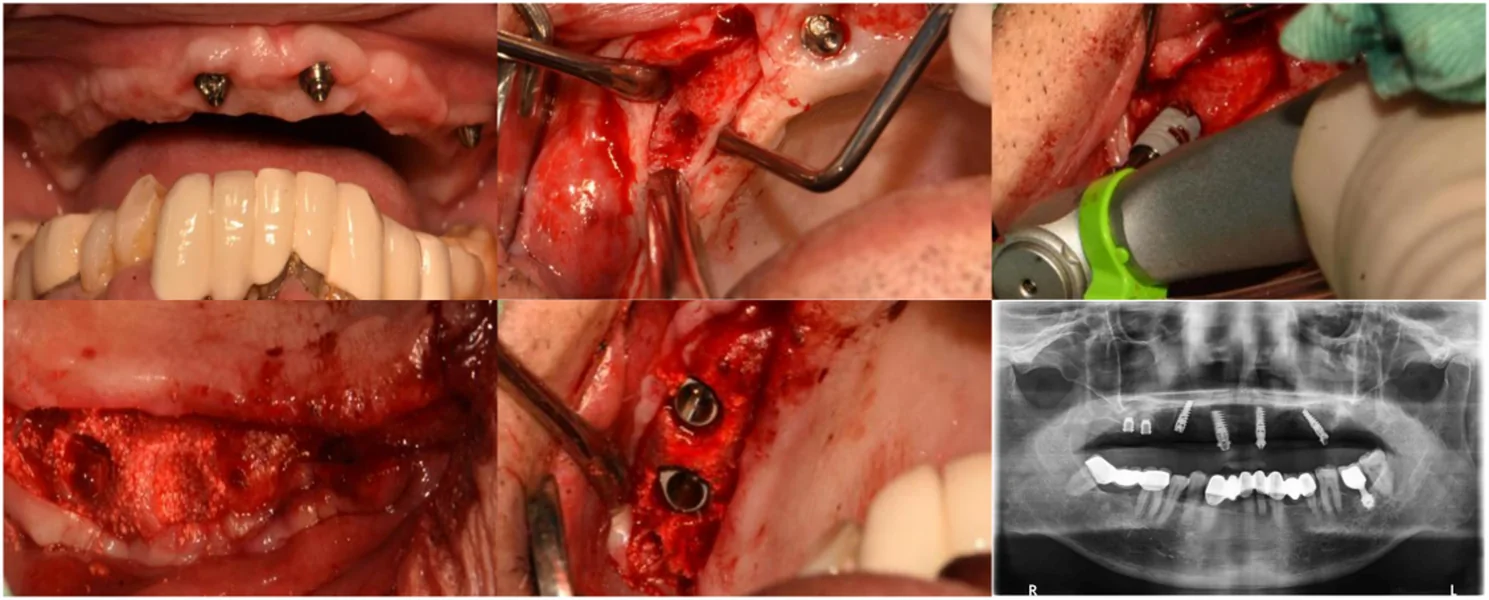
Second-stage operation

Three months after the removal of implant 15, CBCT showed satisfactory bone healing and adequate bone volume at site 15. Due to prior implant failure, the patient desired a safer and more reliable definitive restoration. Specifically, the patient hoped that even if 1–2 implants failed in the future, fixed implant-supported prosthetic restoration of the maxillary dentition could still be achieved without reimplanting new implants. Eventually, the patient agreed to place two additional implants in each of the bilateral molar regions to ensure sufficient “reserve” of implants for future needs. Consequently, the patient and their family declined pterygoid implantation. Given their preferences for lower economic burden, less surgical trauma, and shorter treatment time, the patient and their family opted for transcrestal maxillary sinus lift with simultaneous implant placement. In the event of transcrestal lift failure, lateral window maxillary sinus lift with simultaneous or staged implant placement was designated as the alternative. Implants at sites 15, 16, and 17 were placed by Surgeon B, who has over a decade of clinical experience in implant surgery. For sites 16 and 17, a horizontal crestal incision was made. After flap elevation, the surgical field was fully exposed. Using the Bicon Surgical Kit (Bicon, LLC, Boston, USA), hand-driven reamers and maxillary sinus elevation osteotomes were used alternately to prepare the osteotomy site in a stepwise manner with progressively increasing diameters. The tip of the osteotome was inserted into the socket prepared by the hand-driven reamer, and controlled percussive force was applied to elevate the sinus membrane. The height of internal elevation was referenced based on the scale marks on the bone condenser. After each percussion, the integrity of the maxillary sinus membrane was inspected. The Valsalva maneuver was performed: the patient’s bilateral nostrils were pinched or occluded, and the patient was instructed to breathe out with the mouth closed. The elevated implant socket was visually inspected for the emergence of blood and air bubbles during exhalation. After confirming a negative Valsalva test, bone graft (SynthoGraft, particle size: 0.25–1.0 mm, Bicon, LLC, Boston, USA) was filled into the space between the maxillary sinus floor and the maxillary sinus membrane using a graft delivery system and bone condenser. Two implants (diameter 5.0 mm, length 6.0 mm; Bicon, LLC, Boston, USA) were placed simultaneously. The implant (diameter 4.3 mm, length 13 mm; Nobel Active, Nobel Biocare AB, Sweden) at site 15 was placed at a tilted angle, and three implants underwent submerged healing. The procedure is illustrated in Fig. 4. The postoperative medication protocol was the same as that of the first-stage operation. The patient was instructed to chew on the left side, consume a soft or liquid diet, and was once again emphasized to strengthen oral hygiene maintenance.

#### Management of second implant failure (May 23, 2020)

**Fig. 5:**
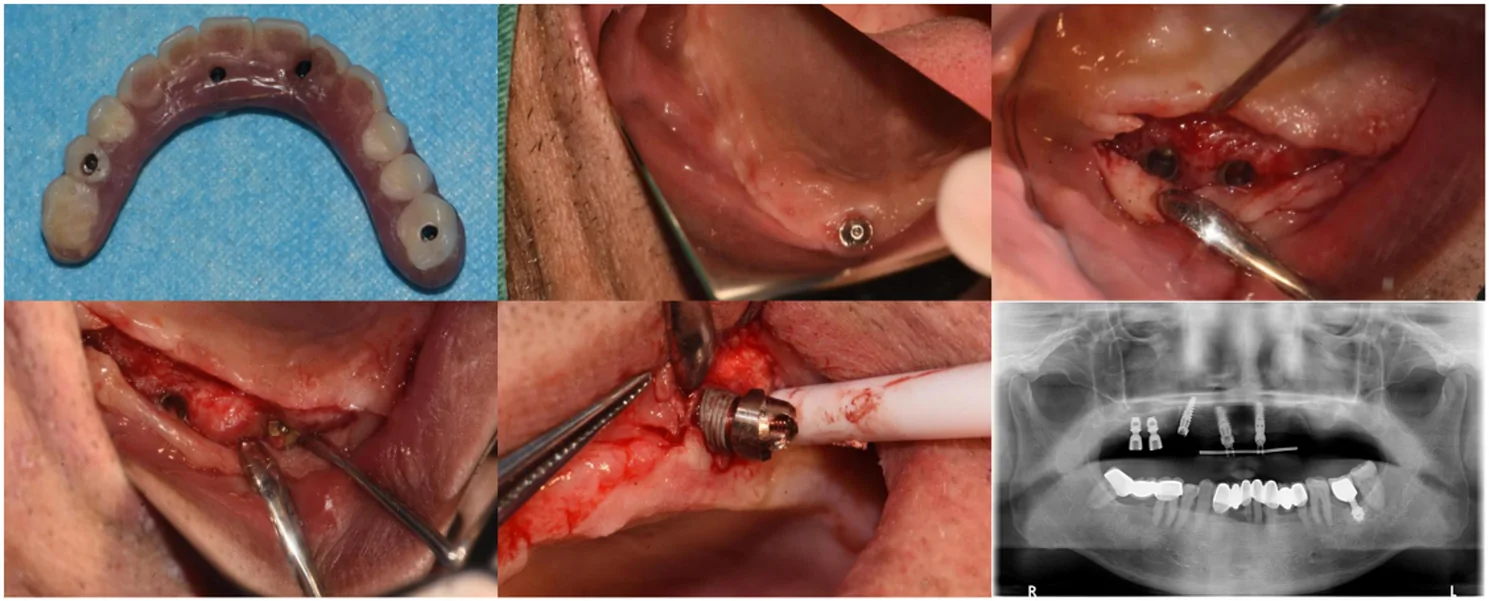
Management of second implant failure

Six months after the second operation, during the preparation for the second-stage surgery (temporary abutment placement) for implants 16 and 17, routine stability assessment of all implants was performed. Implant 25 was found loose, leading to a diagnosis of implant failure. The failed implant 25 was removed simultaneously, the socket was debrided, and the wound was closed, as shown in Fig. 5.

For esthetic reasons, the provisional prosthesis corresponding to sites 13–23 was retained. The occlusal separation between the provisional prosthesis at sites 13 and 23 was greater than 100 μm. The patient was instructed that the provisional prosthesis was primarily for esthetic purposes and did not exert any masticatory function. Additionally, the patient was advised to use auxiliary oral hygiene tools such as an oral irrigator to maintain good oral hygiene.

#### Third-stage operation and final restoration

**Fig. 6:**
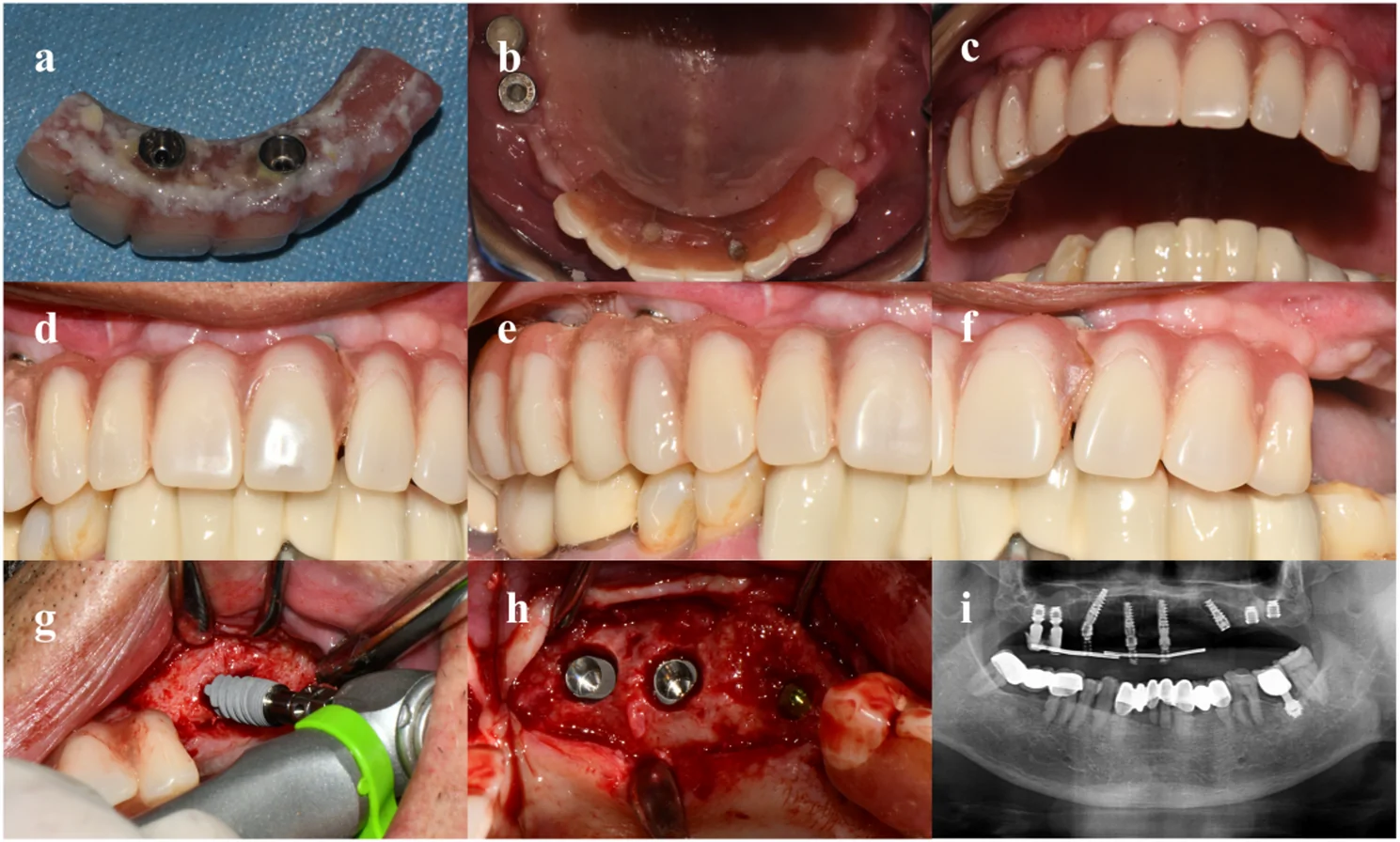
**a**-**b** Poor oral hygiene. **c** Revised provisional prosthesis. **d**-**f** Significant improvement in oral hygiene, with a visible fracture between prosthetic units 21 and 22. **g** Tilted implant placement at site 25. **h** Implant placement at sites 26 and 27 following transcrestal sinus lift. **i** Postoperative panoramic radiograph

Despite multiple rounds of oral health education, the patient still presented with poor oral hygiene after one-month follow-up (June 17, 2020, Fig. 6a–b). The patient reported being unaccustomed to using an oral irrigator and failing to adhere to recommended brushing duration and frequency. The clinician provided prolonged oral hygiene instruction, including the Bass technique, interdental brushes, warm saline rinses, and chlorhexidine gluconate mouthwash twice weekly, emphasizing its critical role throughout the implant treatment continuum. Since the patient resided in another city, weekly telephone follow-ups were conducted to monitor compliance, with family supervision. Notably, oral hygiene significantly improved over a two-month follow-up period (Fig. 6d–f). The Modified Plaque Index decreased from 3 to 1. Cracks were detected in the provisional prosthesis at sites 21–22. The patient was advised to maintain strict adherence to the prescribed oral hygiene protocol. Three months after the removal of implant 25, CBCT showed satisfactory bone healing at site 25. A new provisional prosthesis was refabricated for sites 16–23 and adjusted to achieve group function occlusion to better restore masticatory function (Fig. 6c). One month later, the patient fractured the provisional prosthesis at sites 21–23 due to mastication; the prosthesis was re-restored and adjusted to an occlusion-free state. In accordance with the surgical procedure of the Second-stage Operation, the implant (diameter 4.3 mm, length 13 mm; Nobel Active, Nobel Biocare AB, Sweden) at site 25 was placed at a tilted angle. Transcrestal maxillary sinus lift was performed at sites 26 and 27, with simultaneous placement of two implants (diameter 5.0 mm, length 6.0 mm). The implants at sites 25, 26, and 27 were subjected to submerged healing (Fig. 5g-i). Final fixed prosthodontics was performed in February 2021 (6 months after the third operation). The prosthesis consisted of a zirconia all-ceramic crown and bridge, with occlusal adjustment to centric occlusion.

#### Outcome

**Fig. 7:**
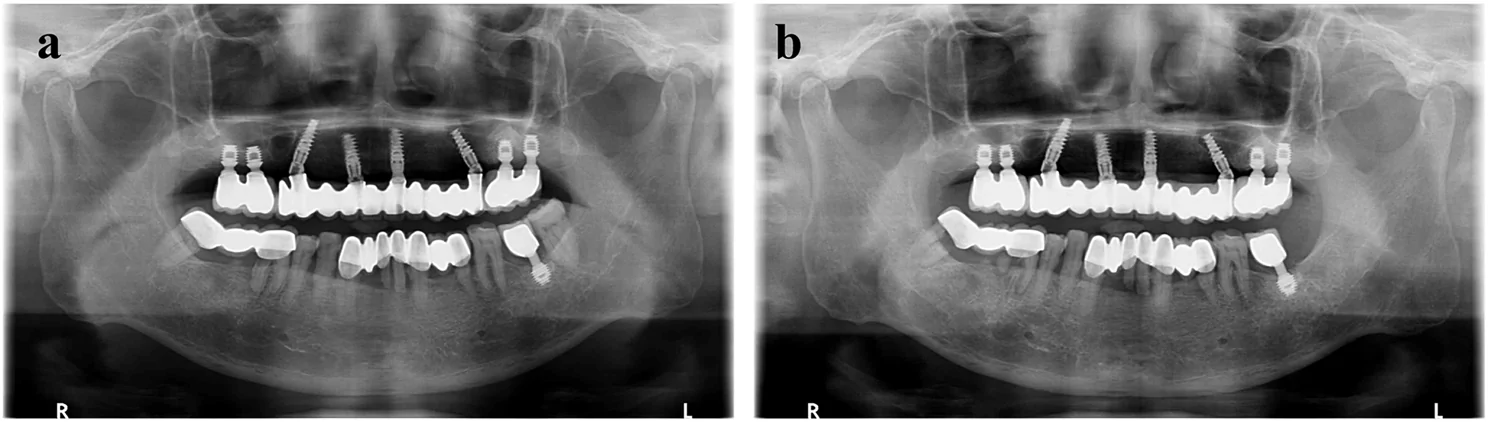
**a** Panoramic radiograph at the time of definitive restoration delivery. **b **Follow-up panoramic radiograph at 55 months

A follow-up examination 3 months after the completion of final restoration showed a stable maxillary prosthesis without loosening or displacement, good occlusal relationship, and a significant improvement in masticatory efficiency (Visual Analog Scale (VAS) score has increased from 2 points before treatment to 8 points after treatment). VAS for masticatory function: 0 = no masticatory function, 10 = normal masticatory function. Intraoral examination revealed no gingival redness, swelling, or bleeding, with a peri-implant probing depth < 3 mm and negative BOP. Radiographic examination showed stable peri-implant bone tissue without obvious bone resorption. As of October 2025, the patient has been followed up for 55 months after the final restoration, with the prosthesis remaining stable and no implant loosening or failure. The patient’s PD symptoms has been well-controlled, oral hygiene has significantly improved compared to pre-treatment, and he is able to perform independent oral cleaning. The patient has expressed satisfaction with the treatment outcome, as shown in Fig. 6. Implant success was evaluated according to the criteria of Buser et al. [9], and all implants have achieved clinical success. The radiographic evaluation was performed using Digimizer Image Analysis Software (V6.2, MedCalc Software bvba, Ostend, Belgium). The changes in implant marginal bone levels were measured on radiographs (a “+ value” indicates an increase, while a “- value” indicates resorption). Taking the long-axis direction of the implants as the reference, the lengths of the four implants on the images were measured three times each, and the average value of all measurements was taken. Calibration was performed based on the actual length (6 mm) and the measured length of the four axial implants in the posterior region, and the mesial and distal bone height changes of each implant were measured separately. The marginal bone changes around the implant after 55 months are as follows: implant 17 (-0.66 mm), implant 16 (-0.41 mm), implant 15 (-0.85 mm), implant 11 (-0.24 mm), implant 21 (-0.42 mm), implant 25 (-0.13 mm), implant 26 (-0.28 mm), implant 27 (+ 0.17 mm) (Fig. 7).

## Discussion

The study is the first to report the management of complications following All-on-4 rehabilitation in a PD patient. This case presents several notable features: the patient was elderly (63 years old) with PD, resulting in poor oral hygiene maintenance ability, which increased the risk of postoperative infection and complications; he had a history of failed implant rehabilitation, with obvious generalized periodontitis and severe alveolar bone resorption, especially extreme bone deficiency in the posterior maxilla (minimum bone height of only 1.2 mm); multiple implant failures occurred during treatment, requiring repeated adjustments to the treatment plan. Such complex cases are not uncommon in clinical practice, and their diagnostic and therapeutic processes provide valuable experience for the management of similar patients.

Combining this case with relevant literature, the main causes of implant failure include:

1. Poor Oral Hygiene: Due to limb tremors caused by PD, the patient initially had inadequate oral hygiene maintenance, leading to peri-implant plaque accumulation, which induced peri-implantitis and ultimately resulted in implant loosening and failure. The core advantage of the All-on-4 technique lies in utilizing sufficient bone volume in the anterior maxilla through tilted implants to avoid complex bone augmentation procedures. However, the survival rate of implants in patients with periodontitis remains controversial. Studies have shown that patients with chronic periodontitis are a significant risk factor for implant loss [10]. In this case, the initial implant failure rate was relatively high (50%, 2/4 implants), which was closely correlated with poor control of periodontal inflammation and inadequate oral hygiene maintenance. The patient also has concomitant xerostomia, which may exacerbate periodontal disease and thereby increase the risk of implant loss.
2. Poor Bone Tissue Condition: Although the bone height at implant sites 15 and 25 was acceptable, long-term periodontitis led to low bone tissue density. Additionally, the thickness of the buccal bone wall at implant site 15 was insufficient, affecting implant stability.
3. Prosthetic Design Defects: During the initial immediate restoration, there was no need to design a cantilever structure at site 16 to meet functional requirements. Patients with PD exhibit high occlusal force and are prone to bruxism at night [11]; the cantilever structure may have also contributed to the failure of the implant at site 15 to a certain extent. After the failure of implant 15, the occlusal contact of the distal cantilever of the temporary prosthesis at site 11 was not promptly eliminated, resulting in excessive force on implant 25 and accelerating its failure.
4. Inappropriate Implant Position: Initially, part of implant 25 was located within the maxillary sinus, failing to obtain adequate bone support and reducing primary stability.

Implant restoration in patients with PD often involves the following challenges: difficulty in performing fine motor skills for oral hygiene maintenance, xerostomia and sialorrhea, occlusal dysfunction, increased incidence of bruxism/clenching, and comorbid potential depression/anxiety [12, 13]. PD itself can cause autonomic nervous system dysfunction and imbalance in the regulation of salivary gland secretion, which, combined with the effects of medications, is more likely to induce xerostomia. Although anticholinergic drugs are the most common cause of drug-induced xerostomia [14], levodopa and dopamine receptor agonists may also indirectly affect salivary secretion. Xerostomia is a common adverse reaction of these drugs [15, 16]. Salivary reduction compromises peri-implant tissue health and induces microbial dysbiosis in the oral cavity. PD patients with xerostomia, particularly those with periodontal disease, are advised to intensify oral hygiene practices, including consistent use of ‌oral irrigators‌ and ‌interdental brushes‌, increased frequency of tooth brushing, and extended brushing duration. Implant-supported prosthesis design should incorporate dedicated cleaning channels or adopt an implant-supported overdenture configuration‌, as these modifications may significantly enhance gingival health by facilitating mechanical plaque removal and reducing biofilm accumulation.

Literature has shown that the implant survival rate in PD patients may be lower than expected, but evidence suggests that the failure occurs in the early rather than late stage [17], indicating that management in the early stage after implant surgery is one of the core priorities. This is consistent with the results of this study; despite frequent implant failures in the short term, the long-term outcome has remained favorable. Despite these numerous challenges, appropriate implant restoration treatment can significantly improve the quality of life of PD patients. Successful restoration not only restores basic masticatory function but also improves nutritional intake, speech clarity, and facial esthetics, thereby enhancing patients’ self-confidence and social participation [14, 18].

In response to these special challenges, researchers have proposed individualized implant restoration protocols. Implant-supported overdentures have been proven to be an effective strategy. They not only facilitate daily maintenance for patients and caregivers but also reduce the risk of mucosal damage caused by patients’ involuntary movements [19]. A case report showed that through this conversion, the patient’s oral hygiene status was significantly improved, and peri-implant inflammation was effectively controlled [20]. The design advantage of implant overdentures lies in their removability, allowing patients and caregivers to perform thorough cleaning, which is particularly important for PD patients with impaired fine hand motor skills. However, fixed implant-supported dentures are often the first choice of patients. ‌Fixed implant-supported prostheses are often the preferred treatment option for patients‌, owing to their stability and absence of removable components. In contrast, impaired swallowing coordination in PD significantly increases the risk of aspiration and dysphagia. Removable implant-supported overdentures, along with their associated components (e.g., clasps, attachments, and acrylic bases), constitute inherent aspiration hazards due to their mobility and detachable design. Fixed implant-supported prostheses, by contrast, present negligible risk of aspiration, as they are rigidly anchored to osseointegrated implants and eliminate mechanical pathways for dislodgement during swallowing. ‌In this study, patient has demonstrated significantly improved masticatory efficiency and high levels of satisfaction after 55 months of functional use of the prosthesis, suggesting that fixed implant-supported prostheses may represent a viable treatment option.

As early as 2015, Liu et al. reported a case of a 76-year-old PD patient who underwent All-on-4 fixed implant restoration in the mandible. Under general anesthesia, the patient received flapless minimally invasive implantation using a NobelGuide digital guide plate; two axial implants (in the lateral incisor region) and two tilted implants (in the first premolar region) were placed in the mandible with an insertion torque of 45–50 Ncm. A provisional prosthesis was placed immediately after surgery (with the cantilever extending only to the second premolar), and fixed restoration was completed after 4 months of osseointegration, with a favorable clinical outcome during 1 year of follow-up [21]. Due to its wide indications, ability to achieve immediate restoration, good esthetics, and a success rate comparable to that of conventional full-arch rehabilitations, the All-on-4 technique has been reported to yield good results in various types of patients in addition to PD patients [22, 23]. For periodontitis patients with comorbid systemic diseases, multidisciplinary evaluation before implant treatment, meticulous intraoperative operation, and long-term postoperative maintenance are crucial [24]. For PD cases, the first step is to strengthen basic periodontal treatment and oral hygiene education. During the subsequent treatment process of this case, repeated oral hygiene education was provided to the patient, who was advised to use a toothbrush, water flosser, and interdental brush for oral cleaning after each meal. Patient compliance is a key factor for implant success, especially in patients with conditions such as PD that affect oral hygiene maintenance. Enhanced health education is necessary, and family assistance with cleaning may be required if necessary. The All-on-4 implant technique is not contraindicated in patients with severe periodontitis, but strict preoperative control of periodontal inflammation is essential. Studies have shown that there is no significant difference in the success rate between 4-implant and 6-implant placement [25]. However, considering the potential higher risk of complications with 4 implants [26], placement of 6 or more implants may be a safer alternative, as it distributes occlusal forces more evenly. For the posterior maxilla, pterygoid implants or maxillary sinus floor elevation techniques are recommended.

Tilted implant placement in the pterygoid process on both sides is also an optional approach. This technique can avoid sinus augmentation surgery and reduce occlusal load by omitting the cantilever structure, with the advantages of minimal trauma and a short treatment cycle [27]. However, in this case, the experience of implant failure led the patient and their family to prefer implanting more implants. The advantage of this approach is that even if one implant fails, the prosthesis can still be installed in a timely manner without affecting the overall restoration effect. Implant restoration is currently developing towards a more minimally invasive direction, and short implants have become a choice with minimal trauma and more predictable clinical outcomes. Implanting short implants in areas with insufficient bone height can avoid complex surgical procedures, shorten the surgical time, reduce costs, expand the indications of implant restoration, decrease postoperative complications, and improve patient satisfaction [28]. Literature has confirmed that 5.0 × 6.0 mm short locking taper implants are sufficient to support single-tooth restoration [29]. In this case, the patient had severe insufficient bone height on both sides; the use of short implants could reduce the elevation height of the maxillary sinus mucosa, thereby lowering the risk of maxillary sinus mucosal rupture. Meanwhile, lateral window maxillary sinus elevation can be used as an alternative option when transcrestal sinus elevation fails, to remedy potential mucosal rupture. The advantages of transcrestal sinus elevation include minimal trauma, small amount of bone graft required, and low economic burden. Some scholars have reported that transcrestal sinus elevation is a feasible option in the severely atrophic posterior maxillary region [30]. In this study, Surgeon B also had extensive clinical experience and research reports related to this technique [31]. However, the transcrestal sinus lift technique carries a risk of implant displacement in severely atrophic posterior maxillary regions [32], and there is currently insufficient evidence-based support for its use. Therefore, it is not recommended as a routine treatment option for severe bone height deficiency in the posterior maxilla. In this case, considering the patient’s wishes, surgical trauma, economic cost, and the clinician’s expertise, the transalveolar maxillary sinus floor elevation with simultaneous implant placement was adopted. Satisfactory outcomes were achieved at sites 16, 17, 26, and 27 using this technique. During surgery, implants should be avoided entering the maxillary sinus, and implants of appropriate diameter and length should be selected to increase the bone contact area. Timely adjustment of the prosthesis is also important: close postoperative follow-up is required to promptly detect and manage issues such as peri-implantitis and occlusal trauma. Once an implant fails, the temporary prosthesis should be immediately adjusted to eliminate unnecessary cantilevers and reduce the impact on remaining implants.

The limitations of this case report include: it is a single case report, and the conclusions need to be verified by more clinical studies; multidisciplinary diagnosis and treatment involving neurology may yield better treatment outcomes; periodontal pathogen detection was not performed, so the direct association between pathogenic bacteria and implant failure cannot be confirmed; the impact of PD medications on implant osseointegration was not evaluated.

## Conclusion

For patients with severe maxillary bone deficiency combined with chronic periodontitis and PD, All-on-4 implant rehabilitation combined with transalveolar maxillary sinus floor elevation can achieve favorable clinical outcomes. The key factors for successful treatment include: adequate preoperative periodontal inflammation control and multidisciplinary assessment; intraoperative optimization of implant position design and meticulous operation; postoperative enhancement of oral hygiene education and establishment of a long-term follow-up mechanism. Patient compliance is an important factor in reducing the incidence of complications, which should be given sufficient attention in clinical practice.

## Data Availability

The datasets used and analysed during the current study are available from the corresponding author on reasonable request.

## Abbreviations

PD: Parkinson’s disease
PLI: Full - mouth plaque index
BOP: Bleeding on probing
CBCT: Cone - beam computed tomography
VAS: Visual Analog Scale
